# Complex Three-Dimensional Co_3_O_4_ Nano-Raspberry: Highly Stable and Active Low-temperature CO Oxidation Catalyst

**DOI:** 10.3390/nano8090662

**Published:** 2018-08-26

**Authors:** Teruaki Fuchigami, Ryosuke Kimata, Masaaki Haneda, Ken-ichi Kakimoto

**Affiliations:** 1 Department of Life Science and Applied Chemistry, Nagoya Institute of Technology, Nagoya 466-8555, Japan; 30411057@stn.nitech.ac.jp (R.K.); kakimoto.kenichi@nitech.ac.jp (K.-i.K.); 2 Advanced Ceramics Research Center, Nagoya Institute of Technology, Gifu 507-0071, Japan; haneda.masaaki@nitech.ac.jp; 3 Frontier Research Institute for Materials Science, Nagoya Institute of Technology, Nagoya 466-8555, Japan

**Keywords:** Catalyst, Co_3_O_4_, complex three-dimensional structure, hydrothermal synthesis, low-temperature CO oxidation, morphological control, nanoparticles, stability

## Abstract

Highly stable and active low-temperature CO oxidation catalysts without noble metals are desirable to achieve a sustainable society. While zero-dimensional to three-dimensional Co_3_O_4_ nanoparticles show high catalytic activity, simple-structured nanocrystals easily self-aggregate and become sintered during catalytic reaction. Thus, complex three-dimensional nanostructures with high stability are of considerable interest. However, the controlled synthesis of complex nanoscale shapes remains a great challenge as no synthesis theory has been established. In this study, 100 nm raspberry-shaped nanoparticles composed of 7–8 nm Co_3_O_4_ nanoparticles were synthesized by hydrothermally treating cobalt glycolate solution with sodium sulfate. Surface single nanometer-scale structures with large surface areas of 89 m^2^·g^−1^ and abundant oxygen vacancies were produced. The sulfate ions functioned as bridging ligands to promote self-assembly and suppress particle growth. The Co_3_O_4_ nano-raspberry was highly stable under catalytic tests at 350 °C and achieved nearly 100% CO conversion at room temperature. The addition of bridging ligands is an effective method to control the formation of complex but ordered three-dimensional nanostructures that possessed extreme thermal and chemical stability and exhibited high performance.

## 1. Introduction

Carbon monoxide (CO), present in exhaust gases from combustion devices (e.g., automotive engines and boilers), poses a hazard to humans. Therefore, the CO created by these combustion devices is converted to carbon dioxide (CO_2_) by an oxidation catalyst. Noble metals such as Pt, Au, and Pd supported on metal oxides show high CO oxidation activity in a wide temperature range [1,2,3,4,5,6,7]. However, noble metals are rare and expensive. Thus, to move toward a sustainable society, developing active and stable catalysts without noble metals is desirable. Among alternative catalysts without noble metals, tricobalt tetraoxide (Co_3_O_4_) has been extensively studied [8,9,10,11]. For low-temperature CO oxidation, the controlled synthesis of nanoscale Co_3_O_4_ has received considerable attention owing to the novel shape/size-dependent properties of nanoscale Co_3_O_4_ [12,13,14,15,16,17,18,19,20,21,22]. For example, L. Hu et al. reported that Co_3_O_4_ nanobelts in which the {110} planes are predominantly exposed achieved 100% CO conversion at around 80 °C [13]. The abundance of active Co^3+^ sites on the {110} plane of Co_3_O_4_ is thought to improve CO oxidation activity at low temperature [16]. In contrast, H. Tüysüz et al. reported that Co_3_O_4_ nanoparticles without predominantly exposed crystal planes showed 100% CO conversion at approximately 30 °C [15]. It is reported that single nano-scale (<10 nm) Co_3_O_4_ nanoparticles showed higher catalytic activity than particles with larger than 10 nm in size [19]. The Co_3_O_4_ nanorods produced by X. Xie et al. exhibited highly stable CO oxidation activity with complete CO conversion at 25 °C for 60 h. However, the conversion rate decreased to 10% after 75 h [14]. The high activities of these Co_3_O_4_ materials are attributed to their nanoscale structures and the abundance of active Co atomic species on the exposed crystal planes. While the effects of nanocrystal size and morphology on catalytic activity have been demonstrated, monodisperse nanocrystals with simple structures are hindered by their low structural stability. The large surface energies of these nanoparticles cause them to self-aggregate and become sintered during reaction owing to atom/ion-diffusion and oxidation-reduction processes. Owing to this aggregation and sintering, the specific surface area decreases, and crystal planes with active sites for catalytic reaction disappear upon long-term use in a current-carrying environment.

Based on the above drawbacks of simple-structured nanocrystals and the relationships between the morphology and CO oxidation activity, we decided to fabricate catalytic nanoparticles with single nano-scale and complex three-dimensional (3D) structures (e.g., nanoflowers, nanocolumns, and sea-urchin like structures) that provide large surface area and high structural stability. Electrode materials with complex 3D structure as sea-urchin like shape maintain their surface structure during reactions and improve long-life charge-discharge properties due to a hierarchical ordered nanostructure and an uneven pattern [23,24,25]. Therefore, we expected that complex 3D structures with the uneven pattern formed via the accumulation of single nano-scale Co_3_O_4_ nanoparticles exhibit high stability and improve catalytic activity in the CO oxidation reaction. Although numerous hydrothermal synthetic methods have been reported for simple zero-dimensional (0D) to 3D nanomaterials, the controlled synthesis of oxide nanoparticles with complex shapes remains challenging because no corresponding synthetic theory has been established. We consider the number of coordination sites on ligands to be a factor in the hydrothermal synthesis of complex 3D nanostructures. Surfactants with one coordination site (e.g., oleic acid, oleylamine, carbonic acid, nitric acid, and acetate acid) are commonly used to generate simple 0D to 3D nanostructures such as nanocubes, nanorods, and nanosheets [16,26,27]. In contrast, surfactants with two or more coordination sites (e.g., urea, phosphoric acid, and oxalic acid) are often used to synthesize complex 3D nanostructures [23,24,25,28,29,30,31,32]. Coordination molecules with one coordination site adsorb on particle surfaces to suppress particle aggregation, particle growth, and the growth of specific crystal planes, resulting in simple structures. Ligands possessing more than two coordination sites might suppress particle growth while promoting nanocrystal self-assembly via particle bridging.

**Figure 1:**
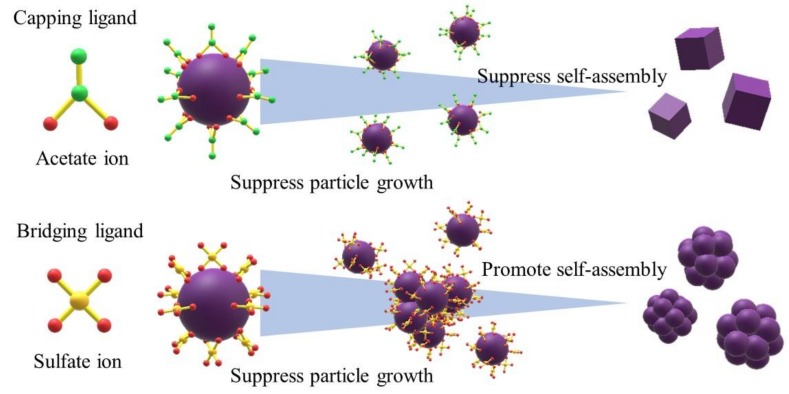
Schematic illustration of the strategy used to synthesize a complex 3D nanostructure using bridging ligands.

Herein, we report the fabrication of complex-shaped 3D Co_3_O_4_ nanoparticles with excellent CO oxidation activity and high stability over a wide temperature range. Such nanoparticles are difficult to produce using conventional methods. Figure 1 demonstrates the strategy we used to control the morphology of the Co_3_O_4_ nanoparticles through the addition of two coordination molecules: sodium sulfate (Na_2_SO_4_) and sodium acetate (CH_3_COONa). The carboxyl group of acetate ion should serve as a coordination site and adsorb on the Co_3_O_4_ nanoparticles. The methyl group of acetate ion should suppress aggregation and particle growth, resulting in simple 0D to 3D nanostructures. The sulfate ions should adsorb to the Co_3_O_4_ nanoparticle surface via one coordination site and inhibit particle growth. The adsorbed sulfate ions could adsorb to additional Co_3_O_4_ nanoparticles via the other coordination site, thereby linking two nanoparticles and promoting self-assembly. The sulfate ions should work as bridging ligands, resulting in an integrated structure composed of Co_3_O_4_ nanoparticles.

Co_3_O_4_ nanoparticles were synthesized via a facile hydrothermal treatment of cobalt ethylene glycolate solution comprising safe and easy-to-use Na_2_SO_4_ and CH_3_COONa. The effects of additional ligands on the morphologies of the resulting nanoparticles were investigated via powder X-ray diffraction (XRD), N_2_ sorption, and high-resolution-transmission electron microscopy (HR-TEM). The addition of sulfate ions resulted in the self-assembly of Co_3_O_4_ nanoparticles with sizes of 7–8 nm, generating particles with raspberry-like morphologies possessing rough surfaces. The CO oxidation activity of the synthesized nanoparticles was evaluated in a fixed-bed continuous-flow reactor as the temperature was decreased from 300 to 25 °C. The surface characteristics and microstructures of the Co_3_O_4_ nanoparticles were characterized via X-ray photoelectron spectroscopy (XPS), HR-TEM, and H_2_ temperature-programmed reduction (H_2_-TPR). The surface nanostructure of the raspberry-shaped Co_3_O_4_ nanoparticles was maintained under catalytic tests in the temperature range of 25–350 °C, and the nanoparticles exhibited and the raspberry-shaped Co_3_O_4_ showed approximately 100% CO conversion at around room temperature. The effects of the morphology, surface characteristics, and microstructure of the Co_3_O_4_ nanoparticles on their catalytic activity are discussed in detail below.

## 2. Experimental Methods

### 2.1. Chemicals

All chemicals were used as received. Co(CH_3_COO)_2_·4H_2_O was purchased from Nacalai Tesque, Inc. (Kyoto, Japan). Ethylene glycol, CH_3_COONa, and Na_2_SO_4_ were supplied by Kishida Chemicals Co., Ltd. (Osaka, Japan).

### 2.2. Synthesis of Precursor and Ligand Solutions

Cobalt glycolate, which served as a precursor, was synthesized as follows [14]. Co(CH_3_COO)_2_·4H_2_O (4.98 g, 0.02 mol) was added to 50 mL ethylene glycol which was already placed in a three-neck round-bottom flask equipped with a magnetic stirring bar. The reaction flask was placed on a mantle heater, and the mixture of Co(CH_3_COO)_2_·4H_2_O and ethylene glycol was heated at 160 °C for 1 h while stirring at 300 rpm. Upon cooling the solution to room temperature under continuous stirring, a homogeneous solution of cobalt glycolate was obtained. The CH_3_COONa solution (0.5 mol·L^−1^) was prepared by adding 8.2 g CH_3_COONa powder (0.10 mol) to 200 mL of distilled water. The Na_2_SO_4_ solution (0.5 mol·L^−1^) was prepared by dissolving 14.2 g Na_2_SO_4_ powder (0.10 mol) in 200 mL distilled water. All solutions were stored at room temperature.

### 2.3. Hydrothermal Synthesis of Co_3_O_4_ Nanoparticles

Distilled water (6.16 mL) and cobalt glycolate solution (2.3 mL) were weighed in a measuring cylinder and then transferred into a 25 mL Teflon container (San-ai Kagaku, Aichi, Japan) equipped with a magnetic stirring bar. Ligands (either CH_3_COONa or Na_2_SO_4_ solution; 1.54 mL) were added to the precursor solution under stirring, and the Teflon container was placed in an autoclave (HU-25, San-ai Kagaku, Aichi, Japan). The autoclave was then heated at 180 °C for 2 h in an aluminum heating unit (HHE-19G-U4, San-ai Kagaku, Aichi, Japan) under autogenous pressure and magnetic stirring at 300 rpm. Subsequently, the autoclave was removed from the heating unit, and the solution was cooled to room temperature under continuous magnetic stirring. The hydrothermally treated solution was then transferred into a 50 mL centrifugation tube and centrifuged at 13,000× *g*. After discarding the clear supernatant, the precipitates were washed with water. This centrifugation and washing process was repeated thrice. Finally, a black powder was obtained by drying at 60 °C for 12 h in air. Co oxide nanoparticles were synthesized using the same procedure but without ligand addition to evaluate the effects of the ligands on the resulting 3D structures.

### 2.4. Characterization

Powder XRD patterns were recorded using a Rigaku MiniFlex600-NB diffractometer (Tokyo, Japan) with Co-Kα radiation, a voltage of 40 kV, and a current of 10 mA. Particle size and morphology were determined via TEM, in a JEM2100F JEOL microscope operating at 200 kV (Tokyo, Japan). The particle size distribution was estimated from the sizes of 100 nanoparticles in the TEM image. N_2_ adsorption isotherms were measured at 77 K in liquid nitrogen in a BELSORP-mini II-CM, MicrotracBEL (Osaka, Japan) after evacuating the samples at 300 °C for 3 h. The specific surface areas of the powders were determined using the Brunauer-Emmett-Teller (BET) method.

Catalytic activity for CO oxidation was evaluated in a fixed-bed continuous-flow reactor. The reaction gas mixture (0.5% CO and 1% O_2_ diluted in He) was fed through the catalyst (30 mg), which had been pretreated in situ by flowing He gas at 300 °C for 0.5 h to remove the ligands adsorbed on the surface of the catalysts. The steady-state catalytic activity was measured every 20 °C as the temperature decreased from 300 °C to 25 °C. The effluent gas was analyzed via on-line gas chromatography with thermal conductivity detection in TCD; Shimadzu C-R8A (Kyoto, Japan). Catalytic activity was evaluated in terms of CO conversion to CO_2_.

The reducibility of the catalysts was evaluated via H_2_-TPR, which was conducted from room temperature to 500 °C under flowing 5% H_2_/Ar (30 cm^3^·min^−1^) at a heating rate of 10 °C·min^−1^. H_2_ consumption was monitored via TCD. The surface compositions of the products were evaluated via Surface Science Instruments XPS, SSX-100 with Al-Kα radiation (λ = 1486.6 eV). 

## 3. Results and Discussion

### 3.1. Synthesis of Raspberry-Shaped Co_3_O_4_ Nanoparticles

**Figure 2:**
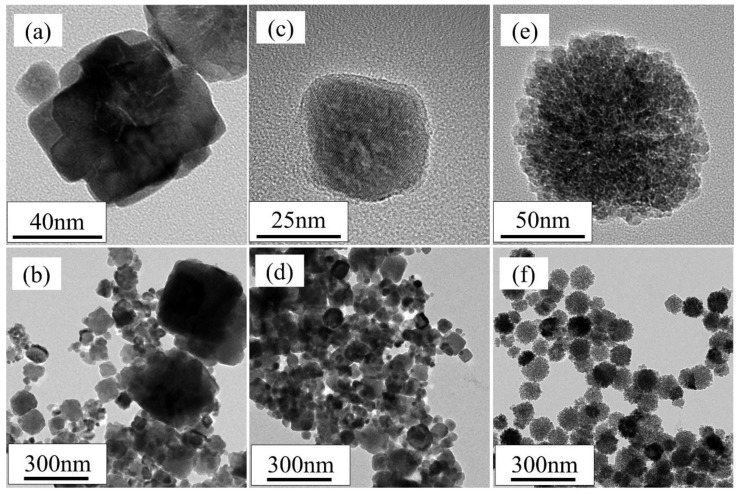
Transmission electron microscopy (TEM) images of Co oxide nanoparticles synthesized (**a**,**b**) without added ligands; (**c**,**d**) with sodium acetate; and (**e**,**f**) with sodium sulfate.

The addition of ligands greatly affected the morphology of Co oxide nanoparticles (Figure 2). Randomly shaped Co oxide nanoparticles were formed by synthesis without added ligands (Figure 2a,b). In addition, the particle size without ligands was relatively large (100–300 nm) and not uniform. The addition of CH_3_COONa to the precursor solution resulted in cube-like shaped particles (Figure 2c,d). Most of these cube-like particles had sizes of 30–50 nm, although a few coarse particles (120 nm) were observed. In contrast, the addition of Na_2_SO_4_ resulted in Co oxide nanoparticles with complex shapes (Figure 2e,f). Co oxide nanoparticles with sizes of 7–8 nm self-assembled into larger spherical particles with rough surfaces and raspberry-like morphologies. As expected, the addition of sulfate ions generated complex 3D Co oxide nanoparticles with surface single-nanoscale structures. The dimeters of the raspberry-shaped particles were approximately 100 nm. The yield of this unique raspberry-shaped nanoparticles was 1 g·L^−1^ and high reproducibility was confirmed by synthesizing them 80 times.

**Figure 3:**
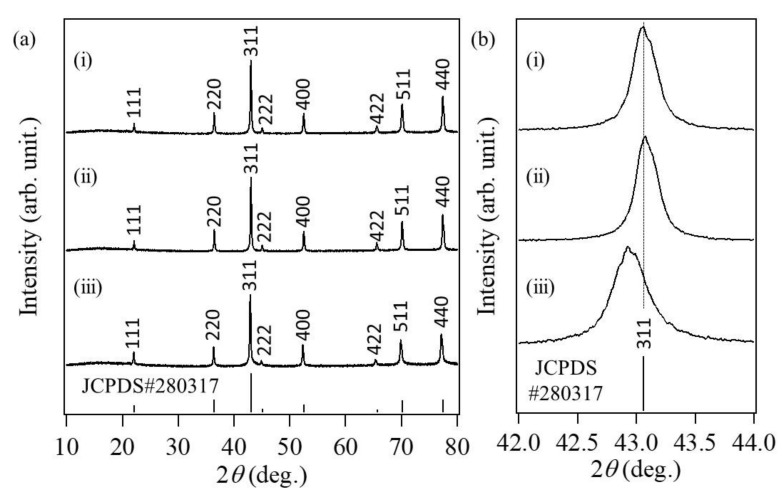
(**a**) Low-angle and (**b**) wide-angle powder X-ray diffraction (XRD) patterns of cobalt oxide nanoparticles synthesized (i) without added ligands, (ii) with sodium acetate, (iii) with sodium sulfate.

Figure 3a shows the XRD patterns of the different synthesized Co oxide particles having different morphologies. All products exhibited typical spinel Co_3_O_4_ crystalline structures (JCPDS no. 280317). The ratio of (220) XRD peak intensity to (400) XRD peak intensity for the raspberry-shaped nanoparticles was 0.963, which is smaller than those of the particles with random morphology (1.10) and cube-like morphology (1.09). Figure 3b shows an enlarged view of the XRD peaks corresponding to the (311) planes of all the synthesized Co_3_O_4_ nanoparticles. Compared with the other Co_3_O_4_ particles, the (311) peak of the raspberry-shaped nanoparticles was broader.

The full width at half maximum (FWHM) values and the structural characteristics of the synthesized Co_3_O_4_ nanoparticles are summarized in Table 1. The FWHM of the raspberry-shaped particles (0.288 deg.) was the smallest among all samples. The crystallite size of the raspberry-shaped particles (54.0 nm) was also the smallest among all samples. These results indicate the raspberry-shaped Co_3_O_4_ particles were composed of smaller Co_3_O_4_ nanoparticles than the other samples. In addition, the XRD peaks of raspberry-shaped Co_3_O_4_ particles were shifted slightly toward a lower angle by approximately 0.1°. This shift is indicative of lattice expansion, which might be explained as follows: (i) the raspberry-shaped particles contained a large amount of oxygen vacancies; and (ii) sulfate ions dissolved interstitially into the crystal structure of the raspberry-shaped particles. 

**Figure 4:**
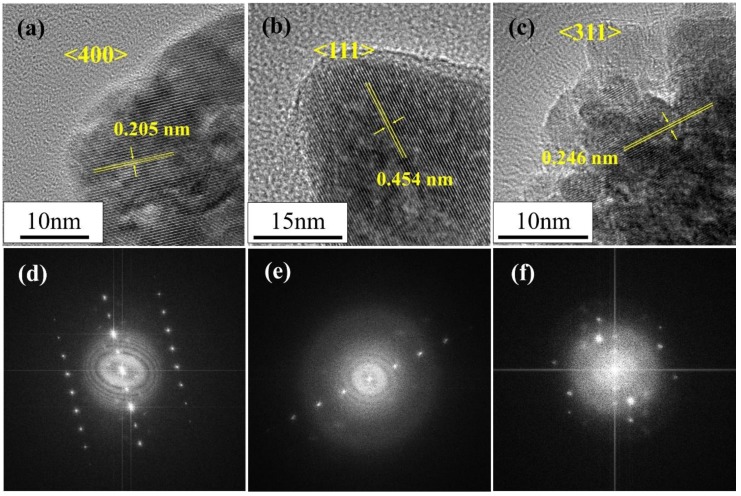
High-resolution transmission electron microscopy (HR-TEM) images and fast Fourier transform (FFT) diffraction patterns of (**a,d**) randomly shaped; (**b,e**) cube-like; and (**c,f**) raspberry-shaped Co_3_O_4_ nanoparticles.

The microstructures of the Co_3_O_4_ nanoparticles were further examined via HR-TEM (Figure 4). The lattice fringe spacings of the randomly shaped and cube-like nanoparticles were 0.205 and 0.454 nm, respectively (Figure 4a,b, respectively), which correspond to the (400) and (111) lattice spacings of typical spinel Co_3_O_4_ (JCPDS no. 280317). The lattice fringe spacing of the raspberry-shaped nanoparticles was 0.246 nm (Figure 4c), which corresponds to the (311) lattice spacing. In addition, the lattice fringes in the TEM image of the raspberry-like particles seem to be uniform within a single particle, indicating that the nano-raspberries comprised single-crystal-like Co_3_O_4_ nanoparticles. The fast Fourier transform (FFT) diffraction patterns of selected areas of the different Co_3_O_4_ nanoparticles are presented in Figure 4d–f. The FFT pattern of the raspberry-shaped nanoparticles indicates vertically aligned single-crystal-like Co_3_O_4_ nanoparticles, in agreement with the above TEM and XRD results. 

**Figure 5:**
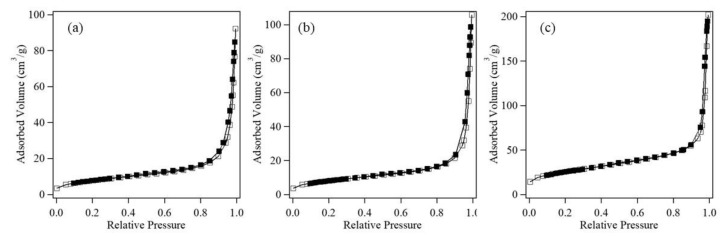
Adsorption isotherms of (**a**) randomly shaped; (**b**) cube-shaped; and (**c**) raspberry-shaped Co_3_O_4_ nanoparticles.

The microstructures of the Co_3_O_4_ nanoparticles were studied based on their adsorption isotherms (Figure 5). The isotherms indicated that the synthesized nanoparticles did not possess pores that would affect their catalytic activity. The BET surface areas are summarized in Table 1. Although the cube-like nanoparticles possessed the smallest average particle size, they also had the smallest specific surface area (29 m^2^·g^−1^) because these particles had relatively smooth surfaces compared to the other Co_3_O_4_ nanoparticles. The uniquely rough surface of the raspberry-shaped nanoparticles resulted in the largest specific surface area of 89 m^2^·g^−1^. The addition of sulfate ions, which have two coordination sites, altered the morphologies of the Co_3_O_4_ nanoparticles to form the unique and complex 3D nanostructure. The roles of the ligands and the formation mechanism of this complex raspberry-shaped nanostructure are discussed as follows. 

The acetate ions contained in the precursor were not sufficient to significantly suppress particle aggregation and the growth of specific crystal planes, resulting in the formation of large, randomly shaped Co_3_O_4_ particles. In contrast, the additional acetate ions supplied from sodium acetate greatly suppressed particle aggregation and particle growth. These acetate ions can be considered to function as typical capping ligands [26,27]. The carboxyl group of acetate ion coordinated with cobalt ions, and the methyl groups exposed on the outermost particle surfaces inhibited the continuous supply of monomer and agglomeration. Thus, relatively small and cube-like nanoparticles were formed. In contrast to acetate ions, sulfate ions have two coordination sites in one molecule and thus function as bridging ligands. In the microporous Yb metal-organic framework synthesized by S. Ma et al. using sulfate ions [33], the two coordination sites of the sulfate ion chelated two metal centers and connected several Yb_4_(μ_4_-H_2_O) secondary building units. In our hydrothermal synthesis, sulfate ions adsorbed to the surfaces of Co_3_O_4_ nanoparticles via one coordination site, where they served as capping ligands to inhibit particle growth by disrupting the supply of monomer. The sulfate ions then bonded to other Co_3_O_4_ nanoparticles via the second coordination site, thus linking two nanoparticles and promoting nanoparticle self-assembly (Figure 1). Based on the crystallite size, the uniform lattice fringes, and the FFT images, the Co_3_O_4_ nanoparticles grew along a specific crystallographic axis, particularly in the case of the raspberry-shaped nanoparticles. These particles were not simply self-assembled; instead, they accumulated while aligning specific crystal planes (e.g., growth by oriented attachment) [34,35].

### 3.2. Catalytic Activity of Co_3_O_4_ Nanoparticles

**Figure 6:**
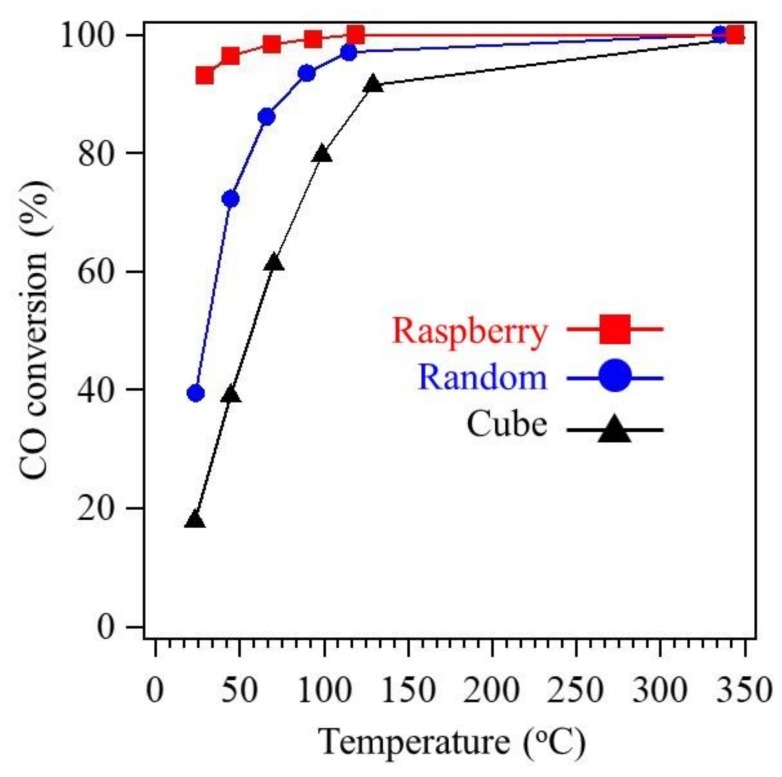
CO conversion efficiency measured with decreasing temperature in the range of 25–350 °C for randomly shaped (blue circles), cube-like (black triangles), and raspberry-shaped (red squares) Co_3_O_4_ nanoparticles.

Figure 6 shows the catalytic activities of the randomly shaped, cube-like, and raspberry-shaped Co_3_O_4_ nanoparticles. The CO conversion was evaluated while decreasing the temperature. Among these samples, the raspberry-shaped nanoparticles provided the best catalytic activity, with almost complete oxidation of CO from 350 to 25 °C. The CO conversion rates of the raspberry-shaped, randomly shaped, and cube-like Co_3_O_4_ nanoparticles at 100 °C were 100%, 90%, and 97%, respectively. The CO oxidation activity of the raspberry-shaped nanoparticles decreased slightly when the temperature decreased below 100 °C; however, these nanoparticles retained a high CO oxidation rate of 93% at room temperature. The CO conversion rates of the randomly shaped and cube-like nanoparticles began to decrease dramatically with decreasing temperature at approximately 100 °C and eventually reached 39% and 18%, respectively. The raspberry-shaped nanoparticles do not have any predominantly exposed crystal planes, and the intensity of the {110} XRD peak was relatively low (Figure 3a). These findings suggest that the exposure of active Co^3+^ sites is not a sole factor in obtaining good CO oxidation activity. The significant differences in CO oxidation rate among the different Co_3_O_4_ nanoparticles cannot be explained by the small differences in specific surface area alone. 

**Figure 7:**
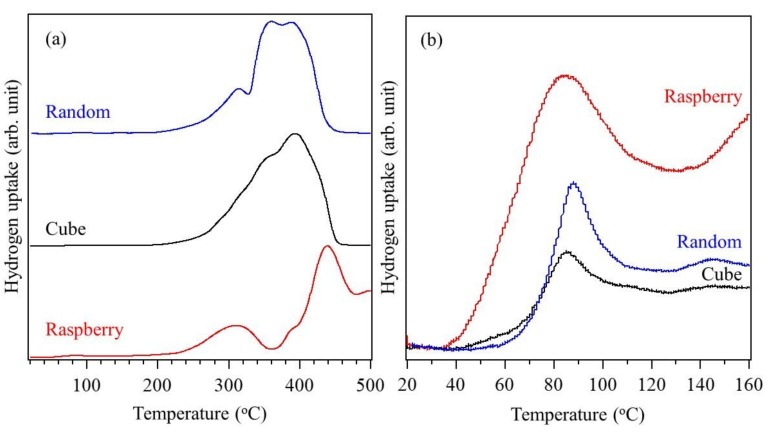
(**a**) H_2_-TPR curves of randomly shaped (blue), cube-shaped (black), and raspberry-shaped (red) Co_3_O_4_ nanoparticles; (**b**) Enlarged view of the H_2_-TPR curves in the temperature range of 20–160 °C.

To further explore the reason for the large observed differences in CO oxidation activity, the surface states of the Co_3_O_4_ nanoparticles, which may affect the CO oxidation activity, were examined using H_2_-TPR (Figure 7). The reduction of large Co_3_O_4_ particles to metallic cobalt has been reported to occur in a single step, while nanoparticles are often reduced via a two-step process [36]. The H_2_-TPR curves of the randomly shaped and raspberry-shaped nanoparticles show broad reduction peaks in the temperature range of 200–340 °C (Figure 7a), corresponding to the reduction of Co^3+^ to Co^2+^ (Co_3_O_4_ to CoO). The peaks in the temperature range of 340–480 °C can be ascribed to the reduction of CoO to metallic Co. The peak at approximately 300 °C was weaker for the cube-like Co_3_O_4_ nanoparticles than for the other samples. This suggests that the relatively rough surfaces of the randomly shaped and raspberry-shaped particles, which have larger surface areas than the cube-like particles, consisted of nano-scale Co_3_O_4_ particles. Iablokov et al. investigated the dependence of CO oxidation activity on particle size and found that the highest reaction rates were obtained by Co_3_O_4_ particles with sizes of 5–8 nm [19]. Additionally, they showed the reaction rates decreased for either smaller or larger sizes. These results suggest that the 7–8 nm nanostructures on the raspberry-shaped Co_3_O_4_ particles are strongly related to their catalytic activity. Figure 7b shows an enlarged view of the H_2_-TPR curves in the temperature range of 20–160 °C. Some small peaks attributed to the reduction of surface oxygen species adsorbed on oxygen vacancies are observed below 160 °C [37]. The reduction peak of the raspberry-shaped particles was larger and began at a lower temperature (29 °C) compared to the randomly shaped and cube-like particles. This finding along with the XRD results (Figure 2b) indicates that the formation of the raspberry-shaped nanoparticles generated a large amount of oxygen vacancies and increased the reactivity of surface-adsorbed oxygen species.

**Figure 8:**
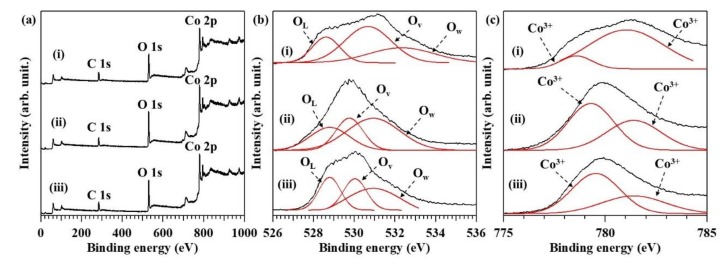
(**a**) Co 2p, O 1s, and C 1s X-ray photoelectron spectroscopy (XPS) spectra; (**b**) O 1s spectra, and (**c**) Co 2p spectra of (i) raspberry-shaped, (ii) randomly shaped, and (iii) cube-like Co_3_O_4_ nanoparticles.

The presence of oxygen vacancies was also investigated by XPS (Figure 8). The ratios of lattice oxygen (O_L_), oxygen vacancy (O_V_), and surface carbonate group (O_W_) were estimated and are shown as red solid lines in Figure 8b. The raspberry-shaped Co_3_O_4_ particles, which had the highest activity for CO oxidation, also had the most oxygen vacancies. The ratio of Co^3+^ to Co^2+^ (Figure 8c) was drastically lower for the raspberry-shaped Co_3_O_4_ particles than for the randomly shaped and cube-like particles. The XPS result indicated Co_3_O_4_ nano-raspberry has less Co^3+^ than Co^2+^. The groups of K. Wang and S. Xie have reported that increasing the content of Co^2+^ can improve low-temperature CO oxidation activity [12,38]. The abundance of oxygen vacancies and Co^2+^ in the raspberry-shaped Co_3_O_4_ particles should promote lattice oxygen mobility and enhance CO oxidation activity [39].

**Figure 9:**
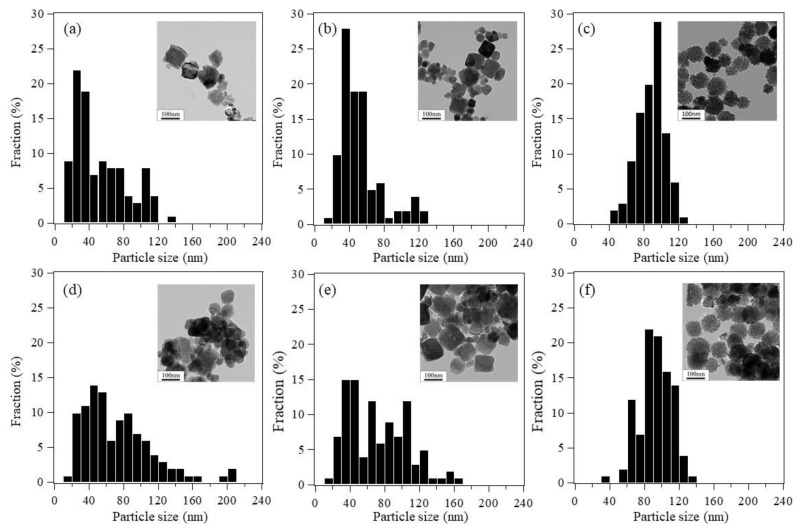
Particle size distributions and TEM images of the (**a**,**d**) randomly shaped; (**b**,**e**) cube-like; and (**c**,**f**) raspberry-shaped Co_3_O_4_ nanoparticles before (**a**,**b**,**c**) and after (**d**,**e**,**f**) the CO conversion tests.

Catalyst particle stability is also an important factor in catalytic activity. Figure 9 shows the particle size distributions and TEM images of the as-synthesized Co_3_O_4_ nanoparticles and the Co_3_O_4_ nanoparticles recovered after the CO oxidation tests. After CO oxidation testing, the number of randomly shaped and cube-like nanoparticles with sizes less than 60 nm decreased, while the number of coarse particles increased as a result of self-aggregation and sintering of the simple-shaped fine particles. Although some of the raspberry-shaped particles with sizes less than 80 nm disappeared after the CO oxidation test, the particle size distribution remained nearly the same after reaction at 350 °C (Figure 9c,f). The crystallite sizes of the Co_3_O_4_ nanoparticles recovered after the CO oxidation tests were calculated using FWHM of (311) peaks shown in Figure S1. The crystallite sizes of randomly shaped and cube-like Co_3_O_4_ nanoparticles increased from 56.5 nm and 61.7 nm to 59.4 nm and 66.5 nm, respectively. In contrast, the crystallite size of the raspberry-shaped Co_3_O_4_ nanoparticles (55.7 nm) after the CO oxidation test was nearly the same as the crystallite size of the as-synthesized raspberry-shaped nanoparticles (54.0 nm). 

**Figure 10:**
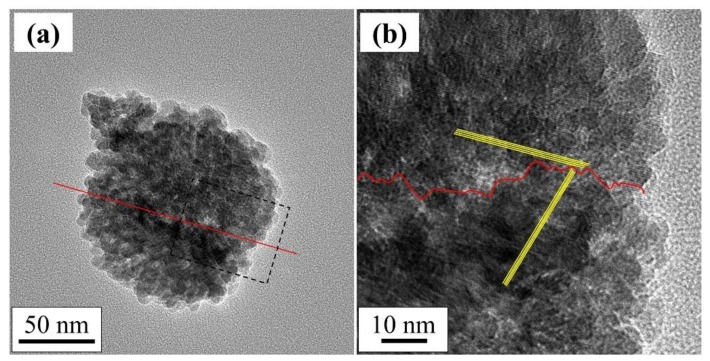
TEM images of (**a**) two raspberry-shaped particles bound via self-aggregation and sintering after CO oxidation testing from 25 to 350 °C and (**b**) enlarged view of the place indicated by dot dashed square in Figure 10a. The boundary between the two particles were indicated by red line and the yellow lines indicates lattice fringes of two raspberry-shaped particles.

Figure 10 shows two TEM images of the raspberry-shaped nanoparticles after CO oxidation reaction. The images show two raspberry-like particles bound via self-aggregation and sintering, where the red solid line indicates the boundary between the two particles. The lattice fringes intersect perpendicularly at this boundary. Interestingly, the rough surfaces of the composed of single nanoscale Co_3_O_4_ particles were maintained after self-aggregation and sintering. This highly stable surface nanostructure might contribute to the complete CO conversion observed over a wide temperature range.

## 4. Conclusions

Raspberry-shaped Co_3_O_4_ nanoparticles were successfully synthesized via a facile hydrothermal method by adding sodium sulfate. The additional sulfate ions function as bridging ligands to promote the agglomeration of Co_3_O_4_ nanoparticles and suppress particle growth, resulting in complex 3D nanostructures composed of Co_3_O_4_ nanoparticles with sizes of 7–8 nm. The synthesized raspberry-shaped nanoparticles have large surface areas (89 m^2^·g^−1^) and exhibit excellent CO oxidation activity (approximately 100% CO conversion to CO_2_ in the temperature range of 30–350 °C). The high oxidation activity of the raspberry-shaped nanoparticles was attributed to the abundance of oxygen vacancies and their unique nanoscale surface morphologies. The surface nanostructure of the raspberry-shaped particles remained stable even under the harsh catalytic reaction process, improving the low-temperature CO oxidation activity. This study demonstrates the facile synthesis of complex 3D metal-oxide nanostructures, which are expected to serve as next-generation materials in catalysis and other applications. This work also describes the effects of coordination molecules on the formation of metal-oxide nanoparticles, which will be helpful to establish a synthesis theory for complex 3D nanostructures.

## Figures and Tables

**Table 1:** Structural characteristics of the Co_3_O_4_ nanoparticles.

Additional Ligand	TEM Observations			BET Surface Area (m^2^·g^−1^)	XRD Measurements	
	Morphology	Primary Particle Size (nm)	Secondary Particle Size (nm)		FWHM (Deg.)	Crystallite Size (nm)
None	Random	60.7 ± 56	-	64	0.269	56.6
Sodium acetate	Cube-like	52.2 ± 25	-	29	0.256	61.7
Sodium sulfate	Raspberry-like	7–8	88.1 ± 16	89	0.288	54.0

## Supplementary Materials

The following are available online at http://www.mdpi.com/2079-4991/8/9/662/s1, Figure S1: XRD patterns of (i) randomly shaped, (ii) cube-like, and (iii) raspberry-shaped Co_3_O_4_ nanoparticles recovered after CO oxidation tests.

## References

[1] Zhang X., Duan D., Li G., Feng W., Yang S., Sun Z. (2018). Monolithic Au/CeO_2_ nanorod framework catalyst prepared by dealloying for low-temperature CO oxidation. Nanotechnology.

[2] Prasad R., Singh P. (2012). A review on CO oxidation over copper chromite catalyst. Catal. Rev..

[3] Royer S., Duprez D. (2011). Catalytic oxidation of carbon monoxide over transition metal oxides. Chem. Cat. Chem..

[4] Haruta M., Tsubota S., Kobayashi T., Kageyama H., Genet M.J., Delmon B. (1993). Low-temperature oxidation of CO over gold supported on TiO_2_, α-Fe_2_O_3_, and Co_3_O_4_. J. Catal..

[5] Haruta M. (2003). When gold is not noble: Catalysis by nanoparticles. Chem. Rec..

[6] Tomita A., Shimizu K., Kato K., Akita T., Tai Y. (2013). Mechanism of low-temperature CO oxidation on Pt/Fe-containing alumina catalysts pretreated with water. J. Phys. Chem. C.

[7] Tomita A., Tajiri K., Tai Y. (2015). Importance of metal-oxide interfaces for low temperature CO oxidation over supported Au and FeO_x_ promoted Pt catalysts. J. Japan Pet. Inst..

[8] Liotta L.F., Wu H., Pantaleo G., Venezia A.M. (2013). Co_3_O_4_ nanocrystals and Co_3_O_4_-MO_x_ binary oxides for CO, CH_4_ and VOC oxidation at low temperatures: A review. Catal. Sci. Technol..

[9] Haneda M., Kawaguchi Y., Towata A. (2017). CoO_x_-FeO_x_ composite oxide prepared by hydrothermal method as a highly active catalyst for low-temperature CO oxidation. J. Ceram. Soc. Japan.

[10] Yao Y.-F.Y. (1974). The oxidation of hydrocarbons and CO over metal oxides: III. Co_3_O_4_. J. Catal..

[11] Wang Y.-Z., Zhao Y.-X., Gao C.-G., Liu D.-S. (2007). Preparation and catalytic performance of Co_3_O_4_ catalysts for low-temperature CO oxidation. Catal. Lett..

[12] Wang K., Cao Y., Hu J., Li Y., Xie J., Jia D. (2017). Solvent-free chemical approach to synthesize various morphological Co_3_O_4_ for CO oxidation. ACS Appl. Mater. Interfaces.

[13] Hu L., Sun K., Peng Q., Xu B., Li Y. (2010). Surface active sites on Co_3_O_4_ nanobelt and nanocube model catalysts for CO oxidation. Nano Res..

[14] Xie X., Li Y., Liu Z.Q., Haruta M., Shen W. (2009). Low-temperature oxidation of CO catalysed by Co_3_O_4_ nanorods. Nature.

[15] Tuysuz H., Comotti M., Schuth F. (2008). Ordered mesoporous Co_3_O_4_ as highly active catalyst for low temperature CO-oxidation. Chem. Commun..

[16] Xie X., Shen W. (2009). Morphology control of cobalt oxide nanocrystals for promoting their catalytic performance. Nanoscale.

[17] Li B., Zhang Y., Du R., Liu L., Yu X. (2018). Controllable synthesis of Co_3_O_4_ nanocrystals as efficient catalysts for oxygen reduction reaction. Nanotechnology.

[18] Song W., Poyraz A.S., Meng Y., Ren Z., Chen S.-Y., Suib S.L. (2014). Mesoporous Co_3_O_4_ with controlled porosity: Inverse micelle synthesis and high-performance catalytic CO oxidation at −60 °C. Chem. Mater..

[19] Iablokov V., Barbosa R., Pollefeyt G., Van Driessche I., Chenakin S., Kruse N. (2015). Catalytic CO Oxidation over well-defined cobalt oxide nanoparticles: Size-reactivity correlation. ACS Catal..

[20] Wang H.-F., Kavanagh R., Guo Y.-L., Guo Y., Lu G., Hu P. (2012). Origin of extraordinarily high catalytic activity of Co_3_O_4_ and its morphological chemistry for CO oxidation at low temperature. J. Catal..

[21] Zhen M. (2014). Cobalt Oxide Catalysts for Environmental Remediation. Curr. Catal..

[22] Yuming D., Kun H., Lin Y., Aimin Z. (2007). A facile route to controlled synthesis of Co_3_O_4_ nanoparticles and their environmental catalytic properties. Nanotechnology.

[23] Liu S., Zhou J., Cai Z., Fang G., Pan A., Liang S. (2016). Nb_2_O_5_ microstructures: A high-performance anode for lithium ion batteries. Nanotechnology.

[24] Lu H., Xiang K., Bai N., Zhou W., Wang S., Chen H. (2016). Urchin-shaped Nb_2_O_5_ microspheres synthesized by the facile hydrothermal method and their lithium storage performance. Mater. Lett..

[25] Niu C., Meng J., Han C., Zhao K., Yan M., Mai L. (2014). VO_2_ nanowires assembled into hollow microspheres for high-rate and long-life lithium batteries. Nano Lett..

[26] Dang F., Mimura K., Kato K., Imai H., Wada S., Haneda H., Kuwabara M. (2012). In situ growth BaTiO_3_ nanocubes and their superlattice from an aqueous process. Nanoscale.

[27] Grandhi G.K., M A., Viswanatha R. (2016). Understanding the role of surface capping ligands in passivating the quantum dots using copper dopants as internal sensor. J. Phys. Chem. C.

[28] Chen H., Lu S., Gong F., Liu H., Li F. (2017). Stepwise splitting growth and pseudocapacitive properties of hierarchical three-dimensional Co_3_O_4_ nanobooks. Nanomaterials.

[29] Ye Y., Chen J., Ding Q., Lin D., Dong R., Yang L., Liu J. (2013). Sea-urchin-like Fe_3_O_4_@C@Ag particles: An efficient SERS substrate for detection of organic pollutants. Nanoscale.

[30] Fuchigami T., Kakimoto K.-i. (2017). Spiky niobium oxide nanoparticles through hydrothermal synthesis. J. Mater. Res..

[31] Fuchigami T., Kakimoto K.-i. (2016). Synthesis of niobium pentoxide nanoparticles in single-flow supercritical water. JPN. J. Appl. Phys..

[32] Yang L.-X., Zhu Y.-J., Li L., Zhang L., Tong H., Wang W.-W., Cheng G.-F., Zhu J.-F. (2006). A facile hydrothermal route to flower-like cobalt hydroxide and oxide. Eur. J. Inorg. Chem..

[33] Ma S., Wang X.-S., Yuan D., Zhou H.-C. (2008). A coordinatively linked Yb metal–organic framework demonstrates high thermal stability and uncommon gas-adsorption selectivity. Angew. Chemie Int. Ed..

[34] Takaaki T., Shuhei F., Yohei T., Kaname Y., Seiji I., Susumu K. (2009). Nanoporous nanorods fabricated by coordination modulation and oriented attachment growth. Angew. Chemie Int. Ed..

[35] Li Z., Sui J., Li X., Cai W. (2011). Oriented attachment growth of quantum-sized CdS nanorods by direct thermolysis of single-source precursor. Langmuir.

[36] Xue L., Zhang C., He H., Teraoka Y. (2007). Catalytic decomposition of N_2_O over CeO_2_ promoted Co_3_O_4_ spinel catalyst. Appl. Catal. B Environ..

[37] Luo J.-Y., Meng M., Li X., Li X.-G., Zha Y.-Q., Hu T.-D., Xie Y.-N., Zhang J. (2008). Mesoporous Co_3_O_4_–CeO_2_ and Pd/Co_3_O_4_–CeO_2_ catalysts: Synthesis, characterization and mechanistic study of their catalytic properties for low-temperature CO oxidation. J. Catal..

[38] Xie S., Deng J., Zang S., Yang H., Guo G., Arandiyan H., Dai H. (2015). Au–Pd/3DOM Co_3_O_4_: Highly active and stable nanocatalysts for toluene oxidation. J. Catal..

[39] Zhang J.J., Wang H.H., Zhao T.J., Zhang K.X., Wei X., Jiang Z.D., Hirano S.I., Li X.H., Chen J.S. (2017). Oxygen vacancy engineering of Co_3_O_4_ nanocrystals through coupling with metal support for water oxidation. ChemSusChem.

