# Supplementary material for: Complex Three-Dimensional Co3O4 Nano-Raspberry: Highly Stable and Active Low-temperature CO Oxidation Catalyst
Source: Nanomaterials (Basel). 2018 Aug 26;8(9):662. doi: 10.3390/nano8090662 (PMC6164048; doi:10.3390/nano8090662)
Supplement: Supplementary file 1 [file nanomaterials-08-00662-s001.pdf]

Supporting information

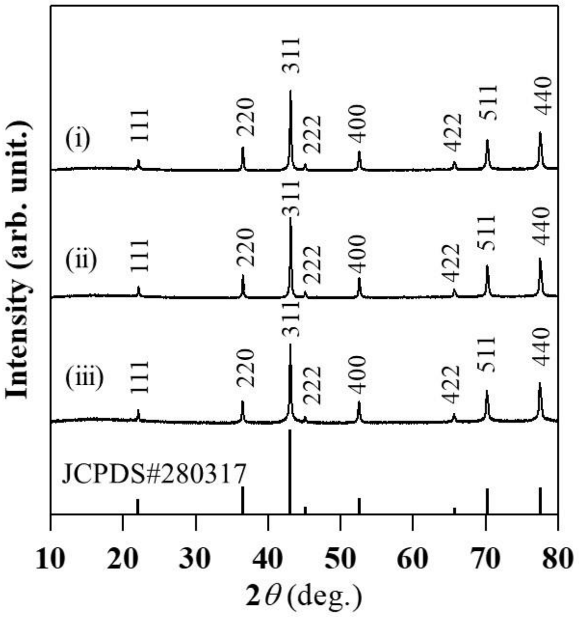

Figure S1. XRD patterns of (i) randomly shaped, (ii) cube-like, and (iii) raspberry-shaped Co_3O_4 nanoparticles recovered after CO oxidation tests.
